# Woman: Her Diseases and Remedies

**Published:** 1851-01

**Authors:** 


### BIBLIOGRAPHICAL NOTICES.

---

Art. V.—*Woman:* her dieases and remedies. A series of letters to his class, by Charles D. Meigs, M. D., Professor of Midwifrey, and the diseases of women and children, in the Jefferson Medical College at Philadelphia, &c. &c. Second edition revised and enlarged. Philadelphia: Lea & Blanchard. 1851.

The merits of the first edition of this work were so generally appreciated, and with such a high degree of favor by the Medical Profession, throughout the Union, that we are not surprised in seeing a second edition of it. It is a standard work on the diseases of females, and in many respects is one of the very best of its kind in the English language.

Upon the appearance of the first edition, we gave the work a cordial reception, and spoke of it in the warmest terms of commendation. Time has not changed the favorable estimate we placed upon it, but has rather increased our convictions of its superlative merits. But we do not now deem it necessary to say more than to commend this work, on the diseases of women, and the remedies for them, to the attention of those practitioners who have not yet supplied themselves with it. The most select medical library would be imperfect without it.

